# Application of the unified protocol for the transdiagnostic treatment of comorbid emotional disorders in patients with ultra-high risk of developing psychosis: A randomized trial study protocol

**DOI:** 10.3389/fpsyg.2022.976661

**Published:** 2022-09-01

**Authors:** Trinidad Peláez, Raquel López-Carrillero, Marta Ferrer-Quintero, Susana Ochoa, Jorge Osma

**Affiliations:** ^1^Parc Sanitari Sant Joan de Déu, Barcelona, Spain; ^2^Investigación Biomédica en Red de Salud Mental (CIBERSAM) Instituto de Salud Carlos III, Madrid, Spain; ^3^Departamento de Psicología y Sociología, Universidad de Zaragoza, Zaragoza, Spain; ^4^Instituto de Investigación Sanitaria de Aragón, Zaragoza, Spain

**Keywords:** anxiety disorders, depressive disorders, ultra high risk for psychosis, unified protocol, transdiagnostic, psychotic disorders, emotional focused therapy

## Abstract

**Background:**

Cognitive Behavioral Therapy is delivered in most of the early intervention services for psychosis in different countries around the world. This approach has been demonstrated to be effective in decreasing or at least delaying the onset of psychosis. However, none of them directly affect the comorbidity of these types of patients that is often the main cause of distress and dysfunctionality. The Unified Protocol for the Transdiagnostic Treatment of Emotional Disorders (UP) is a psychological intervention that combines cognitive-behavioral and third-generation techniques that address emotional dysregulation as an underlying mechanism that these disorders have in common. The application of this intervention could improve the comorbid emotional symptoms of these patients.

**Materials and methods:**

The study is a randomized controlled trial in which one group receives immediate UP plus standard intervention and the other is placed on a waiting list to receive UP 7 months later, in addition to standard care in one of our early psychosis programs. The sample will be 42 patients with UHR for psychosis with comorbid emotional symptoms. The assessment is performed at baseline, at the end of treatment, and at 3-months’ follow-up, and includes: general psychopathology, anxiety and depression, positive and negative emotions, emotional dysregulation, personality, functionality, quality of life, cognitive distortions, insight, and satisfaction with the UP intervention.

**Discussion:**

This will be the first study of the efficacy, acceptability, and viability of the UP in a sample of young adults with UHR. The results of this study may have clinical implications, contributing to improving the model of care for young people who consult for underlying psychotic, anxiety, and/or depressive symptoms that can lead to high distress and dysfunctionality.

**Clinical trial registration:**

[https://clinicaltrials.gov/], identifier [NCT04929938].

## Introduction

In recent decades one of the main topics of research in mental health has been psychotic disorders and, more specifically, their first clinical manifestations. Cumulative research has found evidence highly suggestive of a relationship between the duration of untreated psychosis (DUP) and both short- and long-term prognosis, of a relationship between longer DUP and more severe positive symptoms, and of more severe negative symptoms and lower chance of remission at follow-up (Howes et al., 2021). For this reason, early detection and intervention programs in psychosis have been disseminated in several countries around the world (Taylor, 2016).

In this sense, most psychotic disorders do not appear abruptly. Rather, the onset is preceded by subclinical manifestations of symptoms that gradually increase in frequency and intensity (Shah et al., 2017). This period has been defined and operationalized as “Ultra High Risk” (UHR; Yung et al., 2005). Subsequently, numerous studies have been carried out with the main objective of determining the rates of transition to psychosis as the main measure of outcome (Fusar-Poli et al., 2012). Research has indicated that people that meet UHR criteria are at increased risk of developing psychosis in the short term, and this risk increases over time (Salazar de Pablo et al., 2021). Despite this association, the UHR paradigm is not without criticism. Some previous literature has argued that the “UHR for psychosis” label can be stigmatizing. First, because setting the goal of preventing transition to psychosis/schizophrenia could create a paradox or a self-fulfilling prophecy of failure. Second, because initial research led to clinical trials prescribing antipsychotic medication in the UHR/CHR population (Van Os and Guloksuz, 2017).

There are recent studies that relate the stigma associated with the diagnosis of UHR with a worse prognosis (Colizzi et al., 2020) and stimulated negative stereotypes (Woodberry et al., 2021). For this reason, alternative terms like pre-diagnosis stage’ (PDS), potential of developing a mental illness (PDMI), and disposition for developing a mental illness (DDMI) have been proposed that generate less discomfort among patients and families (Polari et al., 2021a). Generally, pharmacological therapy with antipsychotic medication is not recommended, and psychological interventions represent a more appropriate alternative to offer treatment to people at UHR (NICE, 2014; Orygen The National Center of Excellence in Youth, 2016). In a recent meta-analysis, it was found that early interventions reduced transition rate and attenuated positive psychotic symptoms at 12 months. In addition, psychological interventions demonstrated a significant reduction in transition rates compared to pharmacological therapy (Mei et al., 2021).

Further, transition to psychosis is not the only outcome for people at UHR (Polari et al., 2021b). Some studies have shown different clinical trajectories beyond the transition to psychosis, such as no transition, chronification of attenuated symptoms or their recurrence (Lin et al., 2015), positive improvement, moderate impairment, and severe impairment (Allswede et al., 2020). Another study found 17 different trajectories in UHR patients from complete recovery to transition to psychosis, including no remission, relapse, and recurrence (Polari et al., 2018). A recent study observed that 56.8% of patients with UHR met criteria for a non-psychotic disorder at 6-years’ follow-up (Rutigliano et al., 2016). Similarly, a sample from Spain yielded comparable results (Barajas et al., 2019). Persistence or recurrence of non-psychotic comorbid disorders was associated with worse overall functioning. At baseline, they found that 70.3% of this sample had some comorbidity with a non-psychotic disorder (affective disorder 36.5%, anxiety disorder 10.8%, mixed anxiety-depressive disorder 5.4%, and personality disorder 6.8%). These results showed that although transition to psychosis may be a frequent outcome of patients at UHR, there is also a very high risk of developing another psychiatric disorder (Rutigliano et al., 2016).

Recently, a growing body of literature has suggested that the classification of mental disorders needs to shift from a categorical model (such as DSM or CIE) to a dimensional one (Van Os and Guloksuz, 2017; McGorry et al., 2018). Under this paradigm, it is proposed that there is a continuum between a complete absence of symptoms and severe psychopathology. In this continuum, patients may exhibit symptoms ranging from mild to distressing to indicating the need for specialized help (McGorry et al., 2018), including people meeting the criteria for UHR. This clinical staging and transdiagnostic framework has led to a broader classification of subthreshold risk states (CHARMS) including the bipolar trait vulnerability group, the attenuated (hypo)manic symptom group, the moderate (attenuated) depression group, and the attenuated borderline personality group (Hartmann et al., 2019).

The development of a dimensional approach has fostered the creation of different treatment alternatives, such as transdiagnostic models (Rosellini and Brown, 2019). These models are focused on treating the etiological and maintenance mechanisms shared by different mental disorders (Sauer-Zavala et al., 2017). The Unified Protocol for the Transdiagnostic Treatment of Emotional Disorders (UP; Barlow et al., 2017, 2019) focuses on improving emotional regulation through acceptance, tolerance of intense emotions, and behavior modification (Barlow et al., 2019). To achieve these objectives, it uses traditional cognitive-behavioral techniques (e.g., cognitive restructuring or exposure) combined with third-generation techniques (e.g., mindfulness) (Barlow et al., 2019). This treatment is indicated for people with difficulties in emotional regulation with or without diagnostic comorbidity. Most recently, a clinical trial is under way in Spain to test the efficacy, cost-effectiveness, and implementation characteristics (acceptability, usability, and utility) of a blended intervention which will enhance face-to-face treatment by incorporating an app-based intervention in onsite treatment (Osma et al., 2021).

Current psychological interventions targeting attenuated psychotic symptoms have proved effective in reducing the rates of transition to psychosis in the medium term (Morrison et al., 2004; Van der Gaag et al., 2013b; Ising et al., 2017) and in reducing the severity of psychotic symptoms (Morrison et al., 2012) compared with treatment as usual (TAU). However, further studies are needed to demonstrate more robust results (Van der Gaag et al., 2013b). Cognitive behavioral therapy (CBT) manuals designed to treat UHR (French and Morrison, 2004; Van der Gaag et al., 2013a) do not specifically include comorbid emotional symptoms (affective or anxiety disorders) among treatment goals, although it is known that they are highly prevalent and, in many cases, are the main cause of dysfunctionality (Rutigliano et al., 2016). In the PACE-Manual-Writing Group (Nelson and Orygen Youth Health Research Centre Issuing Body, 2012) there is a module that addresses comorbidity, but in the case of the UP intervention, that is based on the transdiagnostic approach, all psychotherapy techniques have been chosen because they are associated with the core vulnerabilities and processes shared by all emotional disorders (Sauer-Zavala et al., 2017).

The UP has shown a reduction in symptoms of anxiety and depression in several mental disorders (such as major depressive disorder, obsessive-compulsive disorder, and social anxiety, among others) with a large effect size, an increase in adaptive emotional regulation strategies paired with a decrease in maladaptive regulation strategies at a moderate effect size, and an increase in functioning and quality of life (Sakiris and Berle, 2019; Cassiello-Robbins et al., 2020; Carlucci et al., 2021). Furthermore, the benefits of the UP seem to be maintained at 6 months’ follow-up for clinical outcomes and at 12–18 months’ follow-up in functioning (Bullis et al., 2014; Osma et al., 2021). The UP is a standardized and manualized intervention that can be delivered in individual (Barlow et al., 2017) and group formats (Osma et al., 2022). The manual consists of 8 treatment modules.

To date, there is no published study that has used the UP in the treatment of comorbid emotional symptoms in patients that meet UHR criteria, save for a single case study using the UP in a person with treatment-resistant schizophrenia (Grasa and Corripio, 2019) with promising results. There were significant decreases between pre- and post-test measures of anxiety, depressive symptoms, emotional dysregulation, loss of control, rejection, interference scales, and hallucinations, measured with the PSYRATS, as well as a significant increase in quality of life. This work aroused interest in applying the UP to psychotic disorders.

Ultra-high risk (for psychosis) patients often have difficulties in engaging with mental health services (Ben-David et al., 2019) being this situation an important obstacle to receiving appropriate treatment. In addition, young people are often familiar with new technologies (Lupton, 2021) and because of that, we believe that the application of the UP in an online format may reduce the barriers to accessing treatment (Osma et al., 2021). We already know that evidence-based therapies can be administered online without sacrificing their effectiveness (Andersson et al., 2014; McLaren et al., 2021). In a recent review, videoconferencing interventions proved to be reliable and highly acceptable for patients with psychotic disorders (Santesteban-Echarri et al., 2020). With regard to emotional disorders, no significant differences were found in the effectiveness of face-to-face CBT and online CBT. Both of them were shown to be effective in reducing the symptoms of anxiety, depression, and stress, as well as in improving quality of life (Stubbings et al., 2013). The UP has also been shown to be effective in an online format (Carlucci et al., 2021).

A pilot study is under way to assess the feasibility and efficacy of group UP to reduce comorbid emotional symptoms in patients that meet UHR criteria. In the context of the SARS-CoV-2 pandemic, the group sessions are being conducted online. Given the evidence cited above, this method of delivering the intervention would be as effective as in-person treatment with the added benefit of encouraging the attendance of people residing in different geographic territories (Singh and Sagar, 2022).

### Study aims

Given the high level of comorbid anxiety and depression among individuals meeting UHR criteria, the main objective of our study is to evaluate the efficacy of UP in addition to TAU as compared to TAU in targeting symptoms of anxiety and depression in UHR for psychosis patients.

As secondary objectives, changes in attenuated symptoms of psychosis, transition to psychosis rates, cognitive distortions, quality of life, metacognition, personality, and psychosocial functioning, and the satisfaction of participants with the treatment, are being assessed. Further, assessment will be made of whether the results are maintained at 3 months’ follow-up. Different clinical trajectories will be analyzed for comparison with previous studies (Polari et al., 2018).

## Materials and methods

### Study design

This study is a randomized controlled trial. All patients are assessed at baseline. Patients are assigned to either treatment group, one receiving immediate treatment with UP and treatment as usual, or to a waiting list, only receiving treatment as usual. All patients are assessed post-treatment and at 3-months’ follow-up. After the final assessment, patients in the waiting list are offered the UP. A list of random numbers created for this purpose is used.

### Participants

Participants are people who meet criteria of UHR for psychosis and who have comorbid symptoms of emotional disorders and are receiving treatment in one of the Early Pychosis Programs (PIPPEP) in Parc Sanitari Sant Joan de Déu.

The inclusion criteria are (1) age between 18 and 35 years old, (2) a diagnosis of UHR for psychosis in the last 3 years and inclusion in our early intervention program, (3) symptoms of a comorbid emotional disorder, (4) fluent Spanish or Catalan, and (5) signing the informed consent (IC).

The exclusion criteria are (1) a frank psychotic episode in the past or in the present, (2) intellectual disability, (3) an organic disorder that explains current symptomatology.

### Measures

The variables to be studied are evaluated using the instruments described in Supplementary Appendix A.

### Data collection

The evaluation is being carried out at 3 time points. A detailed description of the measures used in each evaluation is reported in Supplementary Appendix B.

Evaluators have been trained in psychological evaluation and specifically in the administration of the CAARMS (Yung et al., 2005). They also are blind to the condition of the study to which the participants have been assigned. In order to ensure internal consistency of the evaluations, interobserver reliability will be calculated with the Cohen kappa. At the beginning of each session with the group receiving UP, two scales are administered, the ODSIS and the OASIS, to measure the severity of depression and anxiety experienced during the previous week, in order to observe fluctuations during treatment. This procedure is performed following the recommendations of the UP manual (Barlow et al., 2019).

CAARMS scores are also collected at the time patients begin treatment in our early psychosis program, prior to entering the baseline assessment of the present study. Medication changes and number or TAU sessions will be recorded as a control variables. Types and dosages of medication will be recorded at the three time points of assessment.

### Interventions

All study participants receive TAU, within our early intervention program. It includes the following interventions: psychological therapy (about 20–40 sessions of CBT) as well as psychiatric treatment (with antidepressants, benzodiazepines and only when needed antipsychotic medication), social work intervention (vocational orientation and support), nursing care (side effects monitoring and healthy habits), individual cognitive remediation (if necessary), and family therapy. The number of sessions received in TAU depends on the clinical status of each patient. The maximum duration of TAU is 5 years. CBT delivered in TAU consists of techniques such as behavioral experiments, socratic questioning, and some exposure techniques mainly focused on subthreshold psychotic symptoms (French and Morrison, 2004; Nelson and Orygen Youth Health Research Centre Issuing Body, 2012; Van der Gaag et al., 2013a). Patients receive weekly or fortnightly sessions of psychotherapy. UP includes establishment of the specific therapeutic aims of each participant, motivation techniques, emotional psychoeducation, teaching functional analysis of the emotional experiences, mindfulness techniques, cognitive flexibility, analysis of emotional behaviors, and training in opposite behaviors. We also use interoceptive exposure, which we never use in individual CBT, and we teach patients to create exposure hierarchies for intense emotions so that they can follow treatment without the continuous supervision of a therapist. The UP consists of 15 online group sessions of 2 h each week. The groups include 5–8 participants. Participants receive an additional follow-up session 1 and 3 months after the end of the program. The sessions work on the 8 modules of UP for the transdiagnostic treatment of emotional disorders, as detailed in the reference manuals (Barlow et al., 2019). A summary of each session of the UP is detailed in Supplementary Appendix C.

Fidelity to the UP treatment protocol is guaranteed through weekly supervision with an accredited therapist. In addition, the therapists who will perform the UP intervention have undergone a 20-h training course. The contents of each module are summarized in an infographic and delivered to patients after the UP session to improve adherence to the intervention and acquisition of the techniques.

Once the participants are recruited to the study, they are randomized into one of the two conditions: TAU + immediate UP (TAU + imm UP) or TAU + WL (TAU + Waiting List). The first group will receive UP immediately in addition to the TAU. The second group will do the TAU while doing the assessments. During this period these patients act as a control group. Seven months later, these patients will receive UP, in addition to TAU. Assessments will also be made at the same time points. This type of study has been carried out previously (Carl et al., 2014). The number of sessions of all the services used in TAU (in both conditions) will be recorded in order to be taken into account when statistical analyses are made. The flowchart of the trial and its different stages is detailed in Supplementary Appendix D.

### Sample size calculation

Accepting an alpha risk of 0.05 and a beta risk of less than 0.2 in a bilateral contrast, 21 subjects in the PU + TAU group and 21 in the WL + TAU group are needed to detect a difference equal to or greater than 1.57 units. The common standard deviation is assumed to be 1.6. A follow-up loss rate of 20% has been estimated.

### Data analysis

To analyze the improvements in primary and secondary variables throughout the study, linear mixed model analysis will be used. This analysis will allow us to study the main effects of time (pre-test, post-test, 3-month follow-up), treatment condition (TAU vs. TAU + UP), and number of sessions received (CBT sessions in TAU, UP sessions, etc.). We will also calculate interaction effects (e.g., treatment condition × time, or treatment condition × number of sessions × time, and the type and dosage of medication) which will reveal whether the treatment condition and the number of sessions received interacted with time in the prediction of changes in study outcomes. In the event that we observe a significant interaction, *post hoc* analysis will be conducted. Due to the nature of the present study, we expect to identify subgroups of patients presenting differing evolutions in study variables according to the number of sessions they have received.

The rate of transition to psychosis of patients in each condition and the CAARMS symptom severity will be calculated in order to evaluate changes in subthreshold psychotic symptoms (Morrison et al., 2012). We will also analyze the different clinical trajectories in the two groups following other previous studies (Polari et al., 2018). If any participant makes a transition to psychosis during their participation in the study, they will be excluded (full-blown psychosis is an exclusion criterion) and their data will be taken into account for further analysis. Finally, satisfaction of patients undergoing group treatment will also be analyzed.

## Discussion

To the best of our knowledge, this is the first study to investigate the effectiveness of UP in a sample of young adults diagnosed with UHR who also have comorbid emotional symptoms. If the results of this study show that UP is effective in treating the comorbid symptoms of UHR for psychosis, this finding could contribute to expanding the psychotherapeutic approaches that can be used with young people presenting with an at-risk mental state. UP may be complementary and/or an alternative to standard CBT approaches. This study would need to be replicated with a bigger sample.

Unified protocol has been shown to be effective in patients with a primary diagnosis of emotional disorders, including cases with comorbidity, according to the systematic review studies and meta-analyses conducted to date (Sakiris and Berle, 2019; Cassiello-Robbins et al., 2020; Carlucci et al., 2021). We hypothesize that this intervention could be equally effective in young patients because there has not been a chronification of their symptoms yet.

The UP contents and the way each emotion regulation technique is introduced and trained for helps patients to normalize their emotional symptoms or disorders, because all people can experience intense emotions and respond with emotional behaviors. It is positive for all people to improve their emotional regulation skills. This perspective focused on training skills can also help to reduce mental health stigmatization. In addition, UP uses expressions like “emotional experiences,” “intense emotions,” and “emotion driven behaviors” instead of other terms like “aggressive response” or “negative emotions.” All these aspects can help reduce the rejection of treatment by mental health services, especially in young people.

The telematic group format of the UP could improve therapeutic adherence in young people, as they are familiar with new technologies (Lupton, 2021). Further, it may encourage the recruitment of patients residing in remote areas and those without specialized care resources.

Cognitive behavioral therapy delivered in TAU is an intervention that has already been shown to be effective in patients of this type (Van der Gaag et al., 2013a). However, significant differences are expected in clinical variables in those patients who additionally receive the intervention with the UP, mainly in comorbid emotional symptomatology.

Given the high comorbidity of emotional disorders in patients with UHR (Rutigliano et al., 2016) and the presence of errors in information processing in both groups of disorders, such as jumping to conclusion, selective care, and catastrophization, as well as avoidant behaviors (Livet et al., 2020), it is likely that these share common transdiagnostic mechanisms with ED. It may be the case that improving emotional regulation will have a positive effect on cognitive biases implicated in the onset and maintenance of both emotional disorders and symptoms of psychosis. If the results show positive associations between improved emotional regulation and improvement in cognitive biases, this would provide a strong theoretical and clinical basis for offering UP to people at the UHR of psychosis.

One limitation of the study is the potential difficulty in isolating the impact of UP on outcome measures. This study is a naturalistic study, that is, it is carried out in the context of public mental health, which is why it is comparable with what has been done up to now. Clinically, it would be more ethical to offer an intervention like UP to all the participants of the study. We expect that the statistical analyses mentioned above can increase the robustness of the study results and solve this limitation.

The UP has already been shown to be effective with similar symptoms (Sakiris and Berle, 2019; Cassiello-Robbins et al., 2020; Carlucci et al., 2021) and this brings us closer to the clinical reality of mental health services for young people in public health. Furthermore, this design may make it easier to collect more samples since this type of patient is not very prevalent. There are several previous studies that have used this type of methodology (e.g., Carl et al., 2014). In this sense, this would be the first step toward obtaining preliminary data on whether the UP adds something to what is already done and to assess aspects of viability and user satisfaction. The next steps will be to compare the UP in isolation with the TAU.

## Ethics statement

This study has been evaluated and approved by the Drug Research Ethics Committee (CEIm) of the Parc Sanitari Sant Joan de Déu. All participants are being provided with an information sheet explaining the objectives and procedure of the study as well as the confidentiality of the data collected. All participants are being asked in writing for their consent in accordance with the Declaration of Helsinki (WMA, 2013) and Law 14/2007 on Biomedical Research.

## Author contributions

TP was the principal investigator of the project, led the development of the manuscript, and investigator in charge of recruiting patients. TP, JO, SO, RL-C, and MF-Q did the study design and decided upon all the outcome measures. MF-Q was carrying out the evaluation process and configured the data collection system. RL-C and TP were carrying out the therapy of the UP and made the infographics of each module. JO was supervising all the therapy process and content of the infographics. SO was responsible for determining sample size and power calculation and proposing all the statistical analyses. RL-C and MF-Q kept the patients linked to the study. JO and SO supervised the development of the study. All authors contributed to the manuscript and approved the final version of the manuscript.

## Abbreviations

UP: Unified Protocol
UHR: Ultra-high Risk (for psychosis)
TAU: Treatment as Usual
ED: Emotional disorders
WL: Waiting list
