# Supplementary material for: Application of the unified protocol for the transdiagnostic treatment of comorbid emotional disorders in patients with ultra-high risk of developing psychosis: A randomized trial study protocol
Source: Front Psychol. 2022 Sep 1;13:976661. doi: 10.3389/fpsyg.2022.976661 (PMC9477005; doi:10.3389/fpsyg.2022.976661)

Supplementary Material

# Supplementary Figures and Tables

Apendix A: Instruments

| Instrument | Construct | Reliability (α) | Response range |
| --- | --- | --- | --- |
|  |  |  |  |
| PRIMARY OUTCOMES |  |  |  |
| BDI-II (Beck et al., 1996; Sanz et al., 2005) | Presence and Severity of Depression | .87 | 4-point Likert scale ranging from 0 (“Absence”) to 3 (“Maximum severity”) |
| BAI (Beck et al., 1993; Sanz et al., 2012) | Presence and Severity of Anxiety | .90 | 4-point Likert scale ranging from 0 (“Not at all”) to 3 (“Severely-it bothered me a lot”) |
| ODSIS (Bentley et al., 2014; Osma et al., 2019a) | Severity of depressive symptoms | .94 | 5-point Likert scale ranging from 0 (“I didn’t feel depressed”) to 4 (”Constant depression”) |
| OASIS (Norman et al., 2006; Osma et al., 2019a) | Severity of anxiety symptoms | .87 | 5-point Likert scale rangin from 0 (“I didn’t feel anxious”) to 4 (“Constant anxiety”) |
| DERS (Gratz et al., 2004; Hervás et al., 2008) | Emotional Disregulation | .93 | 5-point Likert scale ranging from 1 (“Almost never”) to 5 (“Almost always”) |
| PANAS (Watson et al., 1988; López-Gómez et al., 2015) | Positive and Negative Affect | .92 (PA) 0.88 (NA) | 5-point Likert scale ranging from 0 (“Not at all”) to 5 (“Extremely”) |
| SECONDARY OUTCOMES |  |  |  |
| CAARMS (Yung et al., 2005) | Intensity and frequency of subthreshold psychotic symptoms | N.A. | Semi-structured diagnostic interview |
| MINI (Sheehan et al., 1998; Ferrando et al., 1998) | Principal diagnosis of ED | N.A. | Semi-structured diagnostic interview |
| NEO-FFI (Costa et al., 1992; Inchausti et al., 2015) | 5 Personality Factors | .92 (Ex) .64 (Ne) | 5-point Likert scale ranging from 0 (“Strongly disagree”) to 4 (“Strongly agree”) |
| EI (Echeburúa et al., 2000) | Level of affectation of daily life areas by psychological problems | .94 | 6-point Likert scale ranging from 0 (“Nothing”) to 5 (“Very much”) |
| QLI-sp (Ferrans et al., 1985; Mezzich et al., 2000) | Quality of life | .89 | 10-point Likert scale ranging from 1 (“Bad”) to 10 (“Excellent”) |
| CBQ (Peters et al., 2010; Corral et al., 2021) | Presence of 5 cognitive biases (Intentionalising, Catastrophising, Dichotomous thinking, Jumping to Conclusions, Emotional Reasoning) | .87 | 3-point scale between 1 (“absence of bias”) to 3 (“presence of bias”) |
| BCIS (Beck et al., 2004; Gutiérrez-Zotes et al., 2012) | Cognitive Insight | .59 (SR) .62 (SC) | 4-point Likert scale ranging from 0 (“Do not agree at all”) to 3 (“Agree Completely”) |
| TOS (ad-hoc) | Perceived quality of the intervention and its components, usefulness and satisfaction with the online format | N.A. | 10- point Likert scale ranging from 0 (“lowest quality”) to 10 (“highest quality”) |

Note: CAARMS: Comprehensive Assessment of at Risk Mental State; SCID-II: Structural Clinical Interview for DSM, Axis II; MINI: Mini-International Neuropsychiatric Interview; SOFAS: Social and Occupational Functioning Assessment Scale; BDI-II: Beck Depression Inventory-II; BAI: Beck Anxiety Inventory; ODSIS: Overall Depression Severity and Impairment Scale; OASIS: Overall Anxiety Severity and Impairment Scale; DERS: Difficulties in Emotion Regulation Scale; PANAS: Positive and Negative Affect Schedule; PA: Positive Affect; NA: Negative Affect; NEO-FFI: NEO Five Factor Inventory; Ex: Extraversion; Ne: Neuroticism; EI: Escala de Inadaptación (Inadaptation Scale); QLI-sp: Quality of Life Index - Spanish: CBQ: Cognitive Biases Questionnaire; BCIS: Beck Cognitive Insight Scale; SR: Self-Reflectiveness; SC: Self-Certainty

TOS: Treatment Opinion Scale; N.A.: Not Aplicable.

Appendix B: Evaluation schedule

| SCALE | T0: BASELINE | T1: POST-TREATMENT | T2: 3 MONTH FOLLOW-UP |
| --- | --- | --- | --- |
| Socio-demographic questionnaire | X |  |  |
| NEO-FFI | X | X | X |
| BDI-II | X | X | X |
| BAI | X | X | X |
| EI | X | X | X |
| QLI-sp | X | X | X |
| CBQ | X | X | X |
| BCIS | X | X | X |
| PANAS | X | X | X |
| DERS | X | X | X |
| CAARMS | X |  | X |
| MINI | X |  | X |
| ODSIS | X | X | X |
| OASIS | X | X | X |
| OTS |  | X |  |

Appendix C: UP Modules of treatment

| TREATMENT MODULE | GOALS |
| --- | --- |
| 1. Setting goals and motivational enhancement | Increase motivation for change and a sense of self-efficacy in relation to the possibility of change |
| 2. Psychoeducation and registration of emotional responses | Increase knowledge and awareness of the pattern of emotional response |
| 3. Present-focused nonjudgmental awareness | Identify how patients react to emotions and practice the awareness of emotions in the present, without judging them |
| 4. Cognitive flexibility | Learn to identify thought patterns, practice how to modify them, if they are maladaptive, and increase cognitive flexibility |
| 5. Emotional avoidance and behaviours driven by emotions | Learn to identify patterns of emotional avoidance and emotion-driven maladaptive behaviors |
| 6. Emotional awareness and tolerance of physical sensations | Increase of the awareness of the role of physical sensations in emotional experiences by performing exercises that evoke bodily sensations (interoceptive exposure) |
| 7. Interoceptive and situacional exposures | Perform exercises exposure to emotions in daily life situations (interoceptive, live and imaginative) |
| 8. Relapse prevention | Review of the patient's treatment and progress. Programming how to maintain the goals achieved and face difficult situations in the future. |

Appendix D: Flowchart of the study


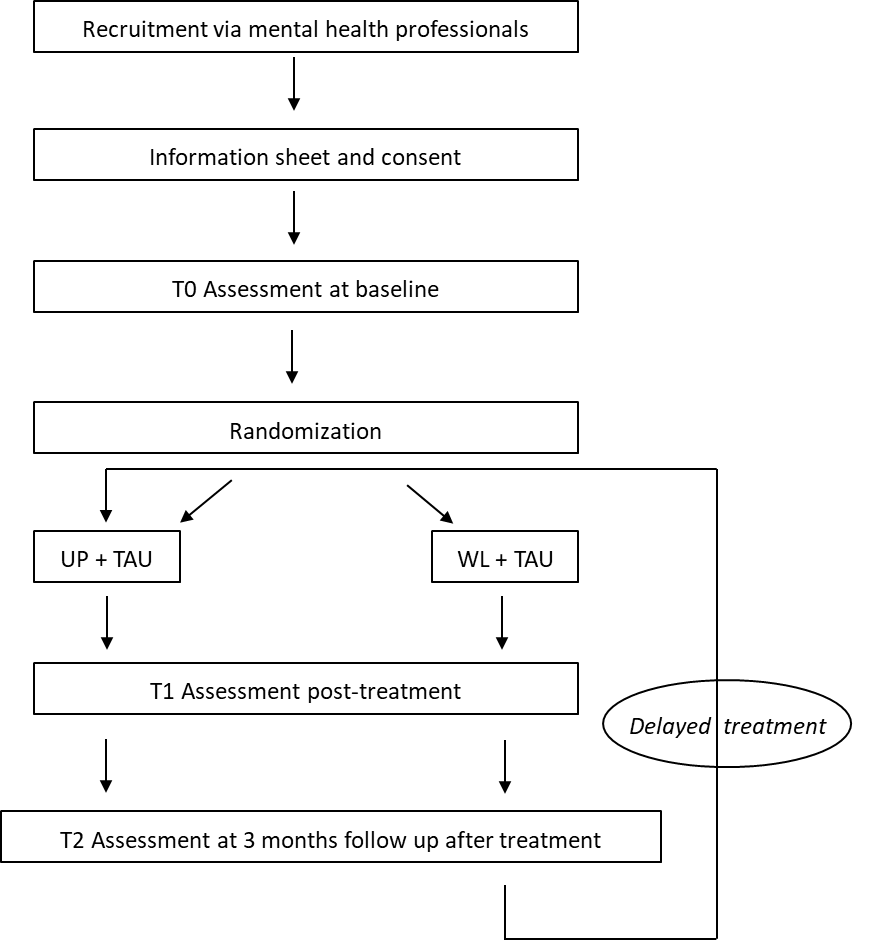

Supplement: Supplementary file 1 [file Table_1.DOCX]
